# From Waste to Health: Landfill Biogas Recovery as a Strategy for Greenhouse Gas Mitigation and Public Health Co-Benefits in Brazil

**DOI:** 10.3390/ijerph23050648

**Published:** 2026-05-13

**Authors:** Estefane Caetano Nazzari, Gredson Keiff Souza, Fernanda Nayara Campos de Almeida, Anderson Rafael Igarashi, Alexandre Diorio, Djeine Cristina Schiavon Maia, Nehemias Curvelo Pereira

**Affiliations:** 1 Department of Chemical Engineering, State University of Maringa, 5790, Colombo av., Building D90, Maringá 87020-900, PR, Braziladiorio2@uem.br (A.D.);; 2 Institute of Chemistry, State University of Campinas, Rua Josué de Castro, s/n, Campinas 13083-970, SP, Brazil

**Keywords:** biogas, green energy, landfill, methane, waste-to-energy

## Abstract

**Highlights:**

**Public health relevance—How does this work relate to a public health issue?**

- Uncontrolled landfill emissions release methane and hazardous trace gases, worsening air quality and climate change.
- Developing countries face elevated health risks due to insufficient waste management infrastructure.

**Public health significance—Why is this work of significance to public health?**

- Controlled biogas recovery reduces population exposure to harmful pollutants, including tropospheric ozone precursors.
- The study demonstrates that landfill gas capture offers measurable health co-benefits alongside renewable energy generation.

**Public health implications—What are the key implications or messages for practitioners, policy makers and/or researchers in public health?**

- Landfill biogas recovery should be integrated into waste management and climate mitigation strategies.
- Expanding gas capture systems supports cleaner air and advances public health goals aligned with SDG 3, SDG 7, and SDG 13.

**Abstract:**

Biogas from municipal solid waste is a promising pathway for renewable energy production while mitigating environmental pollution and public health risks. In this study, biogas emissions from a sanitary landfill in Maringá, southern Brazil, were evaluated using three models (IPCC, LandGEM, and CETESB tool) to estimate methane generation and energy recovery potential. Experimental analysis revealed methane concentrations from 51.10 ± 8.89% to 57.06 ± 1.19% across collection drains, indicating favorable conditions for energy utilization. Methane generation was estimated under different scenarios, reaching up to 1.30 × 10^4^ tonnes of CH_4_, with peak production projected over 25–26 years depending on the model. Beyond energetic relevance, controlled biogas recovery can substantially reduce methane emissions, a key precursor of tropospheric ozone, and limit hazardous trace gas release, improving air quality and reducing population exposure to harmful pollutants. These findings are particularly relevant in developing countries, where insufficient waste management infrastructure leads to uncontrolled emissions, posing elevated environmental and health risks. This study supports integrating landfill biogas recovery into waste management and climate strategies, contributing to Sustainable Development Goals related to clean energy (SDG 7), climate action (SDG 13), and health (SDG 3), demonstrating it as a scalable solution for sustainable urban development.

## 1. Introduction

The global energy transition requires not only the diversification of energy sources but also the mitigation of environmental pollution and its associated impacts on public health [1]. In this context, municipal solid waste (MSW) management plays a critical role, particularly in developing countries where inadequate disposal practices contribute significantly to greenhouse gas (GHG) emissions and local air pollution [2,3].

Landfills are widely used in developing nations as they represent the most cost-effective option for waste management [4]. Furthermore, they constitute one of the largest anthropogenic sources of methane (CH_4_), a greenhouse gas with a global warming potential of approximately 28–34 times higher than carbon dioxide (CO_2_) over a 100-year horizon [5]. In addition to its climate impact, landfill gas contains trace compounds such as hydrogen sulfide (H_2_S) and non-methane volatile organic compounds (NMVOCs), which are associated with respiratory diseases, irritation, and other adverse health outcomes in populations exposed to poorly managed waste disposal systems [6].

NMVOCs comprise a diverse group of substances, such as alkanes, aromatic hydrocarbons, aldehydes, and esters, and are generated from both biochemical degradation processes and the volatilization of organic materials present in waste streams. In landfill environments, NMVOCs originate primarily from the anaerobic decomposition of organic matter and, although generally present at trace concentrations, their environmental and health impacts are relevant since they may interfere with cellular metabolic processes once inhaled and have also been associated with neurotoxic effects and carcinogenic risks. These compounds exhibit high photochemical reactivity and can participate in atmospheric reactions under ultraviolet radiation in the presence of nitrogen oxides, contributing to the formation of tropospheric ozone and photochemical smog. Classified as a secondary pollutant, NMVOCs are linked to adverse health outcomes, including respiratory diseases, increased mortality, and long-term environmental degradation [7]. In this context, beyond methane mitigation, the control of NMVOC emissions from landfills represents an important component of strategies aimed at improving air quality and protecting public health.

In addition, emissions from landfills pose a serious threat to the health of landfill workers and nearby residents, as they contain a large quantity of volatile organic compounds (VOCs) and semi-volatile organic compounds (SVOCs), some of which are mutagenic, carcinogenic, and teratogenic. Based on average pollutant concentrations, carcinogenic risk assessment indicated that health risks in Brazil were predominantly associated with formaldehyde and acetaldehyde, whereas benzene was the principal contributor to carcinogenic risk in Colombia and South Africa [8].

In this context, the need to mitigate emissions from waste management systems while simultaneously meeting energy demands has reinforced the role of waste-to-energy strategies as a sustainable solution. In Brazil, energy demand increases concomitantly with population growth, with an energy consumption per capita of 2.6 to 3.0 MWh/inhabitant/year and an annual estimated average increase of 1.9% until 2031 [9]. Brazil has a predominantly renewable electricity matrix, with hydroelectric power accounting for 47.8%. However, because of this heavy reliance on hydroelectricity, one of the main challenges is its vulnerability to droughts [10]. Over the past decade, the share of hydroelectric power has declined due to environmental restrictions imposed on large-reservoir projects, creating opportunities for hybrid plants that integrate multiple energy sources. This shift has driven investments in alternative renewable sources, such as biomass and biogas (7.4%) and natural gas (7.8%). Additionally, wind and solar energy account for a combined 31.2% of Brazil’s installed capacity for electricity generation [11].

To support the diversification of Brazil’s energy matrix, there is growing interest in utilizing biogas from landfills. Biogas is defined as a gaseous mixture produced through the anaerobic digestion of organic matter, primarily composed of CH_4_ (45–76%) and CO_2_ (30–45%), with smaller amounts of nitrogen (N_2_), oxygen (O_2_), H_2_S, ammonia (NH_3_), and other trace compounds. Methane, its highest constituent, presents a high calorific value, equivalent to 1.15 L of petrol per Nm^3^ of biogas [12,13].

The term biogas is broadly applied to gases generated in different environments, including anaerobic digesters and municipal solid waste landfills, where the resulting gas (named landfill biogas) presents a similar composition and origin, thus being classified as a type of biogas [14]. Moreover, fossil fuels account for only 4.9% of the energy mix, making MSW, particularly its organic fraction, a viable alternative for biogas production and energy generation [15]. Biogas use offers significant economic and environmental advantages, including a reduction in GHG emissions and the valorization of biomass [16].

Electricity consumption in Brazil is, approximately, 475,648 GWh, with the South region accounting for 87,906 GWh, or about 18.5% of the country’s total [17]. Additionally, Brazil generates 79 million tonnes of MSW annually, most of which is disposed of in landfills, contributing to increased GHG emissions, including the release of biogas [18].

In Brazil, public policies have increasingly recognized the importance of methane mitigation and waste-to-energy conversion strategies. The “PNRS—Política Nacional de Resíduos Sólidos” (National Policy for Solid Waste) established guidelines for environmentally appropriate waste disposal and encourages energy recovery from landfill biogas [19]. Recently, the “Programa Metano Zero” (Zero Methane Program) has promoted the reduction in methane emissions across key sectors, including solid waste management [20]. At the international level during COP-2026, Brazil is a signatory of the Global Methane Pledge (2021) [21], which aims to reduce global methane emissions by at least 30% by 2030.

Economic incentives for low-carbon energy have also been strengthened through the “RenovaBio” program, which established a market for decarbonization credits (CBIOs), potentially including biomethane derived from biogas [22]. In addition, the ANP Resolutions n° 685/2017 and n° 886/2022 define quality standards for biomethane, enabling its injection into natural gas grids and use as a vehicle fuel, thus enhancing the commercial viability of biogas upgrading technology [23,24].

Despite this regulatory and policy framework, landfill biogas remains an underutilized renewable energy source in Brazil, particularly in medium-sized municipalities, where infrastructure limitations hinder the implementation of gas capture and energy recovery systems. Its potential for bioenergy generation is significant due to the high methane content, as methane has a high calorific value (~9.5 kWh/Nm^3^) [13,16]. This gap represents a missed opportunity to simultaneously address climate change, energy security, improve air quality, and reduce environmental health risks.

Therefore, this study aims to evaluate the biogas production potential of the Maringá city sanitary landfill using established modeling tools, IPCC (Intergovernmental Panel on Climate Change) and LandGEM (Landfill Gas Emissions Model), while also discussing the implications of methane mitigation and energy recovery for environmental health and sustainable management policies. For this, key factors such as annual MSW volume, waste composition and biodegradation rates were considered. This approach offers an integrated framework that combines site-specific experimental data, multi-modeling simulation and a quantitative discussion of public health co-benefits, providing a comprehensive assessment of landfill biogas recovery in developing countries.

## 2. Materials and Methods

### 2.1. Landfill Location and Characteristics

The city of Maringá is in the Northwestern region of Paraná State, South region of Brazil, with its sanitary landfill at a latitude of 23°25′31″ S and longitude of 51°56′19″ W. The landfill was opened in 1974 as an open dump and was subsequently transformed into a sanitary landfill in 2006 due to environmental and regulatory requirements. Currently, the facility receives waste from 14 municipalities. The landfill handles an average of 350 tonnes of MSW per day, corresponding to approximately 0.81 kg per capita per day. In 2025, the total amount of waste managed reached approximately 138,900 tonnes, comprising 117,860 tonnes of household waste, 6889 tonnes of recyclable materials, 1145 tonnes of bulk waste, 8959 tonnes of construction and demolition waste, and 4049 tonnes of street sweeping residues.

**Figure 1:**
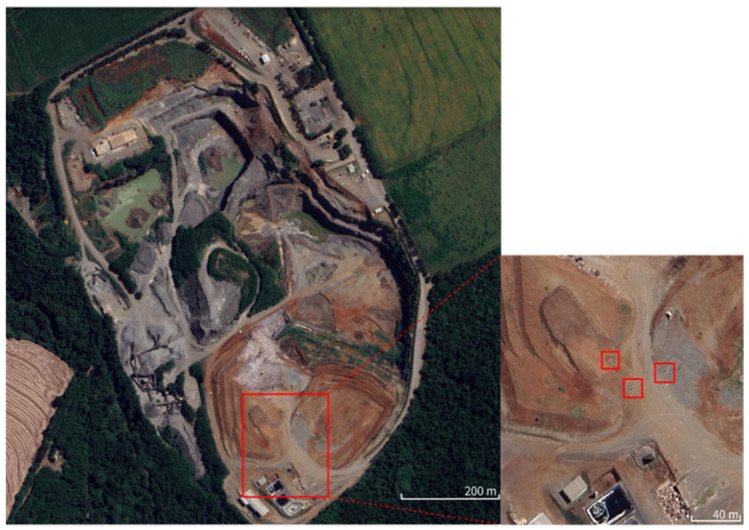
Municipal landfill of Maringá and biogas collection drains. From left to right, drains 1, 2 and 3. Source: Google Earth, 2025 [25].

The landfill is divided into areas called cells 1 and 2, with areas of 25,995.86 m^2^ and 37,999.09 m^2^, respectively. Biogas samples were collected in a timely manner in three separate vertical drains, as shown in Figure 1. The analyses of the biogas samples were carried out at the Laboratory of Separation Processes and Particulate Systems (LPS) of the Chemical Engineering Department (DEQ) at the State University of Maringá (UEM).

### 2.2. Modeling for Estimating Biogas in the Landfill

Biogas production at the Maringá landfill was estimated using models proposed by the Intergovernmental Panel on Climate Change (IPCC) and LandGEM. These methodologies are commonly employed in the design of projects aimed at reducing GHG emissions in sanitary landfills under the Clean Development Mechanism. Both methods are based on first-order decay kinetic equations and share methane generation potential (L_0_) as a key parameter.

The methane generation potential depends on the composition of the waste, particularly the amount of degradable material, known as degradable organic carbon (DOC). DOC represents the fraction of organic carbon available for biochemical decomposition by bacteria [26].

To determine L_0_ and DOC, according to the IPCC method [26], Equations (1) and (2) were applied:

(1) $$L_{0}\;=\;\mathrm{MCF}\;\times \;\mathrm{DOC}\;\times \;{\mathrm{DOC}}_{f}\;\times \;F\;\times \left(\frac{16}{12}\right)$$

in which MCF—methane correction factor; DOC—degradable organic carbon (tonnes of organic carbon/tonne of MSW); the difference DOC_f_—DOC is the fraction decomposed under anaerobic conditions; F—fraction of CH_4_ by volume generated in the landfill gas.

(2) DOC=(0.40 × A)+(0.17 × B)+(0.15 × C)+(0.3 × D)

in which A—fraction of paper/cardboard and textile waste; B—fraction of waste from gardens/parks; C—fraction of food scraps; D—fraction of wood waste.

The DOC_f_ is the fraction of carbon considered to be slowly degradable, as is the case of lignin, calculated using Equation (3):

(3) $${\mathrm{DOC}}_{f}\;=\;\left(0.014\;\times \;T\right)\;+\;0.28$$

in which T is the temperature in the anaerobic zone of both landfills and dumps, estimated at about 35 °C, which lies within the 20 °C to 65 °C range required for biological activity in landfills [27,28].

To convert the L_0_ values obtained in Equation (1) from kg of CH_4_ /kg of MSW to m^3^ of biogas/tonne of MSW, it was necessary to divide the result by the methane density (0.0007168 tonnes/m^3^) [29].

The L_0_ constant was calculated according to the IPCC model and subsequently applied to the LandGEM model estimates.

#### 2.2.1. Model IPCC

The software developed by the IPCC established methods for inventories of greenhouse gases for various sectors of energy, waste, and agriculture, being free to download, use, and reproduce [30]. The methodology proposed by the IPCC [31] considers the input of waste data such as quantity, composition, and kinetic parameters.

To estimate methane emissions from landfills, the IPCC model [31] was used as described in Equations (4)–(12):

(4) $$\mathrm{DDOCm}\;=\;{\mathrm{DDOCm}}_{0}\;\times \;e^{-k_{1}\;\times \;t}$$

(5) $${\mathrm{DDOCm}}_{\mathrm{decomp},\;t}={\mathrm{DDOCm}}_{0}\;\times \;\left(e^{-k_{1}\;\times \;(t-1)}-e^{-k_{1}\;\times \;t}\right)$$

(6) $${\mathrm{DDOCmd}}_{t}=W_{t}\;\times \;\mathrm{DOC}\;\times {\;\mathrm{DOC}}_{f}\;\times \;\mathrm{MCF}$$

(7) $${\mathrm{DDOCm}}_{\mathrm{rem},\;t}=\mathrm{DDOCm}d_{t}\;\times \;e^{-k_{1}\;\times \;(13-M)/12}$$

(8) $${\mathrm{DDOCm}}_{\mathrm{dec},t}=\mathrm{DDOC}{\mathrm{md}}_{t}\;\times \;\left[1-e^{-k_{1}\;\times \;(13-M)/12}\right]$$

(9) $${\mathrm{DDOCma}}_{t}=\mathrm{DDOC}m_{\mathrm{rem},t}+(\mathrm{DDOC}{\mathrm{ma}}_{t-1}\;\times \;e^{-k_{1}})$$

(10) $${\mathrm{DDOCm}}_{\mathrm{decomp},\;t}=\mathrm{DDOC}m_{\mathrm{dec},t}+\mathrm{DDOC}{\mathrm{ma}}_{t-1}\;\times \;(1-e^{-k_{1}})$$

(11) $${{\mathrm{CH}}_{4}}^{\mathrm{generated},\;t}=\mathrm{DDOC}m_{\mathrm{decomp},t}\;\times \;F\;\times \left(\frac{16}{12}\right)$$

(12) $${{\mathrm{CH}}_{4}}^{\mathrm{emission},t}=\left[\sum_{x}{{\mathrm{CH}}_{4}}^{\mathrm{generated},x,t}-R_{t}\right]\times \;\left(1-{\mathrm{OX}}_{t}\right)$$

in which t—inventory year; x—fraction of material/waste category; W_t_—mass of waste deposited in year t; MCF—methane correction factor; DDOCm—mass of DDOC that will decompose under anaerobic conditions at time t; DDOCm_0_—mass of DDOC when the reaction starts; DOC—degradable organic carbon (anaerobic conditions) in the year of deposition; DOC_f_—fraction of DOC decomposable under anaerobic conditions; DDOC—degradable organic carbon decomposable under anaerobic conditions; DDOCmd_t_—mass of DDOC deposited in year t; DDOCm_rem,t_—mass of DDOC disposed in year t that remains undecomposed at the end of the year; DDOCm_dec,t_—mass of DDOC disposed in year t that has decomposed by the end of year t; DDOCma_t_—mass of undecomposed DDOC accumulated at the end of year t; DDOCma_t−1_—total mass of undecomposed DDOC accumulated at the end of year t − 1; DDOCm_decomp,t_—mass of DDOC decomposed at the end of year t; CH_4_^generated,t^—CH_4_ generated in year t; F—fraction of CH_4_ by volume in the generated landfill gas; 16/12—molecular weight ratio CH_4_/C; CH_4_^emission,t^—CH_4_ emitted in year t; R_t_—CH_4_ recovered in year t; OX_t_—oxidation factor in year t; k_1_—biodegradation rate constant; M—month when the reaction starts (delay time +6 months).

The data suggested by the model for each type of material consider the fraction of degradable organic carbon (DOC), methane correction factor (MCF), biodegradation constant (k_1_), and gravimetric composition of the municipality of Maringá—PR.

Obtaining values for DOC is based on the composition of the waste and the amount of carbon available in each component of their mass, and the MCF values change due to the form of waste disposal that is used, categorized as shown in Table 1.

**Figure 2:**
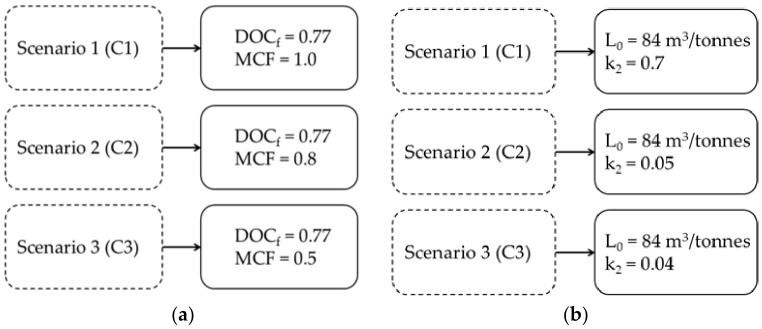
(**a**) Scenarios for MCF using the IPCC model; (**b**) Scenarios using LandGEM.

Estimates for methane emission were simulated in three waste management scenarios, varying different landfill projections and considering the different methane correction factors, as shown in Figure 2 for the IPCC model and the LandGEM model.

For data entry in the software, we considered the average methane concentration collected in the field from the biogas, the degradable organic carbon (DOC) content, and the biodegradation constant (k_1_).

The degradation constant (k_1_) is related to the time for the DOC to decay in relation to its initial mass. The values for this parameter suggested by the IPCC [31] range from 0.035 to 0.40, as shown in Table 2. For the methane biodegradation constant, the decay was performed for both scenarios, using the parameter k_1_, which has different values for each waste category.

#### 2.2.2. Model LandGEM

The LandGEM model was implemented on a Microsoft Excel spreadsheet, which is available for download at the EPA’s Clean Air Technology Center [32]. This methodology is employed to estimate the amount of total landfill gas emissions, including methane, carbon dioxide, non-methane volatile organic compounds (NMVOCs), and individual air pollutants from MSW landfills.

The equation used for the first-order decomposition rate to estimate annual emissions during a given period established by the data compiler is described in Equation (13):

(13) $$Q_{{\mathrm{CH}}_{4}}=\;\sum_{t=1}^{n}\sum_{j=0.1}^{1}{k_{2}L}_{0}\left(\frac{M_{i}}{10}\right)e^{{-k_{2}t}_{\mathrm{ij}}}$$

in which Q_CH4_—annual methane generation in the year of calculation (tonnes/year); i—1 year time increment; n—(year of calculation)—initial year of waste acceptance; j—time increment of 0.1; k_2_—methane generation rate (year^−1^); L_0_—methane generation potential (m^3^/tonne); M_i_—mass of waste accepted in the i-th year (tonne); t_ij_—age of the j-th section of the M_i_ residue mass accepted in the i-th year (decimal years, e.g., 3.2 years).

The simulations were performed by applying standard parameters suggested by the model and input parameters based on real data from the landfill. The estimates of the LandGEM model [33] were also made considering three scenarios; however, the parameter used was the methane generation rate (k_2_). The analyzed data vary according to the scenarios, as can be seen in Figure 2b.

The methane generation rate (k_2_) was determined from the mass of waste deposited in the landfill. As the methane generation rate increases, it results in a higher k_2_ value, however, it decreases over time. The value of the methane generation constant is affected by several factors, among them: the humidity of the residues, the availability of nutrients for microorganisms, pH, and temperature [31]. The k_2_ values were obtained from field measurements based on US landfill sites. However, they range from 0.003 to 0.21, depending on the climate of the region [33].

The methane generation potential is a consequence of the composition of the waste deposited in the landfill: the higher the cellulose content of the waste, the higher its value. These values range from 6.2 to 270 m^3^/tonne of MSW [33]. The applied values of k_2_ and L_0_ in LandGEM can be seen in Table 3. As proposed in LandGEM, landfill gas is assumed to be composed of 50% methane and 50% carbon dioxide, in addition to other elements of NMVOCs and atmospheric pollutants. Other amounts of methane can be used, provided data are available, but it is not advisable to use ranges outside of 40% to 60% [33].

### 2.3. Measurement of Volume and Composition of Biogas

The temperature and volumetric flow of biogas were measured in vertical drains, with the same frequency as the samples collected, as mentioned above. The measurement was performed in a passive ventilation system, without the use of compressors or other forced suction equipment.

The flow rate was determined using a hot wire anemometer (model 42-Testo). In the drains, an opening was made for the insertion of the equipment, thus obtaining the average speed (m/s) of the gas flow and its temperature (°C).

To calculate the flow, the diameter of the tubes and the measured velocity were used, thus determining the volumetric flow of biogas, as described in Equation (14):

(14) $$Q\;=\;\left(v\;\times \;A\right)\;\times \;\frac{273.15}{273.15\;+\;T}\;\times \;P$$

in which Q is the biogas flow rate (Nm^3^/s), v is the biogas velocity (m/s), A is the area of the gas passage section (m^2^), T is the biogas temperature (°C), and P is atmospheric pressure (bar).

The barometric pressure considered during the measurements was obtained from the Main Climatological Station of Maringá (ECPM), in agreement with the National Institute of Meteorology (INMET). The meteorological stations managed by INMET consider the following parameters: atmospheric pressure, temperature, relative humidity, precipitation, solar radiation, wind direction, and speed [34]. The analyses were performed at different times of the day, considering the average pressure during the day.

To determine the volumetric composition of the biogas, we used a gas chromatograph (GC)—model Thermo Scientific Trace GC Ultra (Thermo Scientific, Waltham, MA, USA), equipped with a Thermal Conductivity Detector (TCD) and a Flame Photometry Detector (FPD), fitted with a HP-Plot U column (30 m × 0.53 mm × 0.20 μm; Agilent Technologies, Santa Clara, CA, USA), using helium as carrier gas. The gases of interest were as follows: CH_4_, CO_2_, H_2_S, O_2_, and N_2_. The injection was performed using the splitless method, with a sample volume of 1 mL, at a temperature of 70 °C, with a duration of 10 min for each analysis.

GC calibration was performed using standard samples with known gas compositions. From the chromatograms obtained in the injections of the standards, it was possible to determine the composition of the biogas. 

The determination of the composition and percentage of each biogas component is related to the peak area and retention time. Correction factors proposed for the components of interest were used for each area produced in the peaks. The areas were corrected and normalized, thus defining the molar concentration of the biogas components.

### 2.4. Landfill Electrical Generation Estimate

The software “Biogas, energy generation and use—landfills, version 1.0” [35], similarly to LandGEM, is based on a first-order kinetic equation, estimating biogas generation and energy recovery. In addition, it considers the following properties: landfill characteristics, estimation of biogas generation in the landfill, available energy and use, choice of energy use technology, simplified estimate of the price of biogas use, carbon dioxide price, generation, and printing and storage of the report. In this study, the use of the software is limited only to the estimation of the electric energy potential.

The software proposed by São Paulo State Environmental Company (CETESB) [35] was used to estimate the energy available per year, calculated from Equation (15):

(15) $$P_{x}\;=\;\frac{Q_{x}\;\times \;P_{c\;(\mathrm{methane})}}{31,536}\;\times \;E_{c}\;\times \;\frac{k}{1000}$$

in which P_x_—power available each year (kW); Q_x_—methane flow each year (m^3^ CH_4_/year); P_c(methane)_—calorific value of methane, equal to 35.53 × 10^6^ (J/m^3^ CH_4_); E_c_—user-reported gas collection efficiency (%), 31,536.000 = 1 year (s/year); k = 1.000 (dimensionless).

## 3. Results

### 3.1. Landfill Characteristics

The L_0_ value obtained for the sanitary landfill was determined according to the IPCC method [26], based on Equation (1). For the estimation, the gravimetric composition values of the MSW of the city of Maringá—PR were used, as described by PMM [36]. Using the method proposed by the IPCC, an L_0_ value of approximately 84 m^3^ CH_4_ per tonne of MSW was obtained. The value of L_0_ was also used in LandGEM modeling. For both models, a methane fraction of 55% was considered, according to the average value obtained from the analysis carried out in the field for the city landfill.

### 3.2. Modeling Results

**Figure 3:**
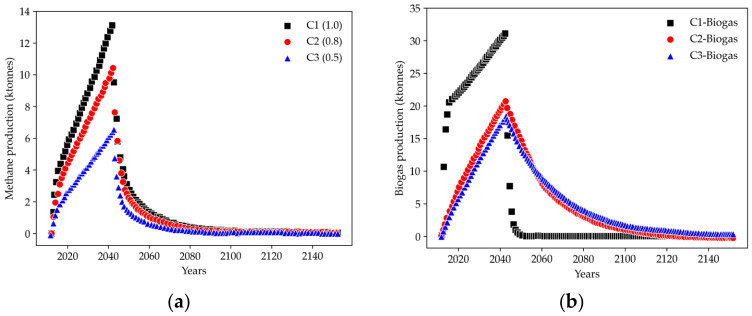

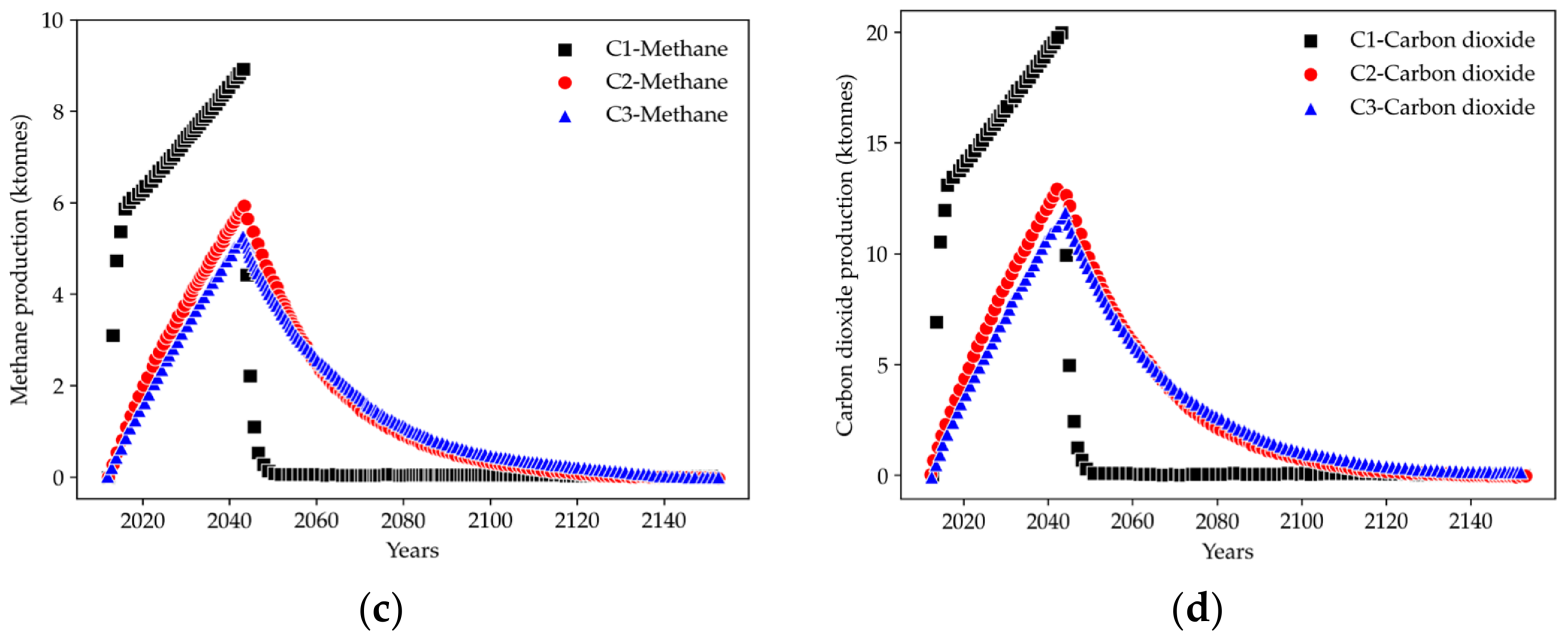
(**a**) Methane production according to the IPCC model; (**b**) biogas production according to the LandGEM model; (**c**) methane production according to the LandGEM model; (**d**) CO_2_ production according to the LandGEM model.

The outcomes from the application of the IPCC model and LandGEM are presented in Figure 3. In these simulations, three scenarios (C1, C2, and C3) were defined to represent different management conditions and parameter sets for methane generation. Each scenario was evaluated to determine the production of biogas and its main constituents (CH_4_ and CO_2_) over the years.

#### 3.2.1. Results of the IPCC Model

The different scenarios analyzed using the IPCC method represent various practical approaches to waste management. Figure 3a presents the estimated methane production considering three MCF values: 1.30 × 10^4^ tonnes CH_4_ (Scenario 1—MCF 1.0), 1.04 × 10^4^ tonnes CH_4_ (Scenario 2—MCF 0.8), and 6.54 × 10^3^ tonnes CH_4_ (Scenario 3—MCF 0.5), all reaching maximum production in the year 2042. From the landfill’s opening after renovations in 2012 until the complete degradation of the waste mass, the total methane production is estimated at 3.14 × 10^5^ tonnes CH_4_ (C1), 2.57 × 10^5^ tonnes CH_4_ (C2), and 1.60 × 10^5^ tonnes CH_4_ (C3). Of these totals, 2.38 × 10^5^ tonnes CH_4_, 1.91 × 10^5^ tonnes CH_4_, and 1.19 × 10^5^ tonnes CH_4_ are generated within the first 30 years for scenarios 1, 2, and 3, respectively, corresponding to approximately 75% of total methane production in all analyzed scenarios.

#### 3.2.2. The LandGEM Model

The simulations using LandGEM are based on different values of the methane generation rate. Figure 3b shows the different scenarios for biogas production. An estimated biogas production of 3.11 × 10^4^ tonnes/year (C1), 2.06 × 10^4^ tonnes/year (C2), and 1.84 × 10^4^ tonnes/year (C3) was observed, with maximum peaks in the year 2043, one year after the end of MSW disposal. The total production of biogas from 2012 until the degradation of the material deposited in the landfill can generate 7.95 × 10^5^ tonnes/year (C1), 7.68 × 10^5^ tonnes/year (C2), and 7.64 × 10^5^ tonnes/year (C3), of which 7.34 × 10^5^ tonnes/year, 3.46 × 10^5^ tonnes/year, and 2.99 × 10^5^ tonnes/year correspond to the years 2012 to 2042 for scenarios 1, 2, and 3, respectively.

Methane production using LandGEM, as shown in Figure 3c, reached its peak in the year 2043, with maximum values of 8.90 × 10^3^ tonnes/year (C1), 5.91 × 10^3^ tonnes/year (C2), and 5.27 × 10^3^ tonnes/year (C3). The total CH_4_ production was 2.28 × 10^5^ tonnes/year (C1), 2.20 × 10^5^ tonnes/year (C2), and 2.19 × 10^5^ tonnes/year (C3), where 2.10 × 10^5^ tonnes/year, 9.91 × 10^4^ tonnes/year, and 8.57 × 10^4^ tonnes/year are for scenarios 1, 2, and 3, respectively.

As shown in Figure 3d, the production of carbon dioxide was 2.00 × 10^4^ tonnes/year (C1), 1.32 × 10^4^ tonnes/year (C2), and 1.18 × 10^4^ tonnes/year (C3), all with maximum production in the year 2043. The total production of CO_2_ was 5.11 × 10^5^ tonnes/year (C1), 4.94 × 10^5^ tonnes/year (C2), and 4.91 × 10^5^ tonnes/year (C3), with 4.71 × 10^5^ tonnes/year (C1), 2.23 × 10^5^ tonnes/year (C2), and 1.92 × 10^5^ tonnes/year (C3) corresponding to production between the years 2012 and 2042.

For all scenarios and compounds analyzed using the LandGEM model in the period of 30 years, it was observed that the total production is approximately 92% (C1), 45% (C2), and 40% (C3), which corresponds to the years that the landfill is in operation with daily provisions.

### 3.3. Results of the Measurement of Volume and Composition of Biogas

**Figure 4:**
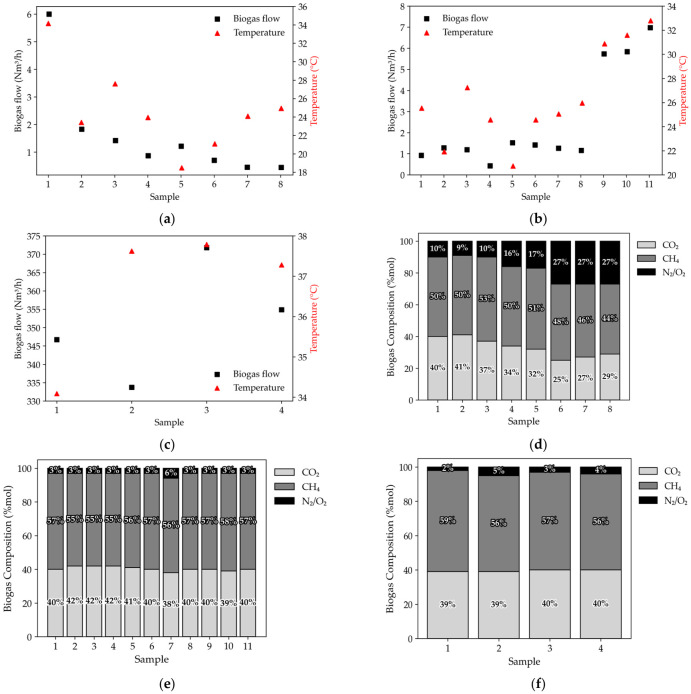
(**a**) Biogas flow—Drain 1; (**b**) Biogas flow—Drain 2; (**c**) Biogas flow—Drain 3; (**d**) Biogas composition—Drain 1; (**e**) Biogas composition—Drain 2; (**f**) Biogas Composition—Drain 3.

The volumetric flow of biogas was obtained by measuring the velocity and temperature of the biogas at the exit of the drains, being mainly related to temperature and disposal time, as represented in Figure 4.

Figure 4a shows the observed flow rate from drain 1, resulting in an average of 1.63 ± 1.83 Nm^3^/h of biogas. The strong influence of temperature on biogas production can be observed.

In Figure 4b, it is possible to observe the biogas flow to drain 2, where the average was 2.82 ± 2.44 Nm^3^/h.

Figure 4c shows the flow rate of drain 3, representing an average of 3.52 × 10^2^ ± 15.89 Nm^3^/h, as this area receives waste daily, in addition to the indicated temperatures being above 34 °C. The three analyzed drains present an average flow of approximately 1.19 × 10^2^ Nm^3^/h.

Figure 4d–f show the compositions determined for the biogas collected in the drains present at the site.

The average concentrations of gases collected at the landfill in drain 1 (Figure 4d) were 14.19 ± 14.92% for O_2_ + N_2_, 51.10 ± 8.89% for CH_4_, and 34.69 ± 6.09 for CO_2_, with variations in the constituents of the biogas over time. The high concentrations of N_2_ + O_2_ observed in drain 1 in a few days were due to the logistics of the landfill. The drain in question had undergone structural changes and was later eliminated, which caused greater external interference during the collections.

In Figure 4e,f it is possible to observe that the concentrations analyzed in drains 2 and 3 were 3.19 ± 1.01% for O_2_ + N_2_, 56.40 ± 1.02% for CH_4_, and 40.380 ± 1.15 for CO_2_ and 3.39 ± 1.08% for O_2_ + N_2_, 57.06 ± 1.19% for CH_4_, and 39.53 ± 0.74 for CO_2_, respectively. The concentration remained constant during the analyzed period, indicating that the degradation of residues is in the methanogenic phase. These rates were maintained until a large amount of the available substrate was consumed by microorganisms [37].

### 3.4. Electrical Generation Estimate from the Landfill

**Figure 5:**
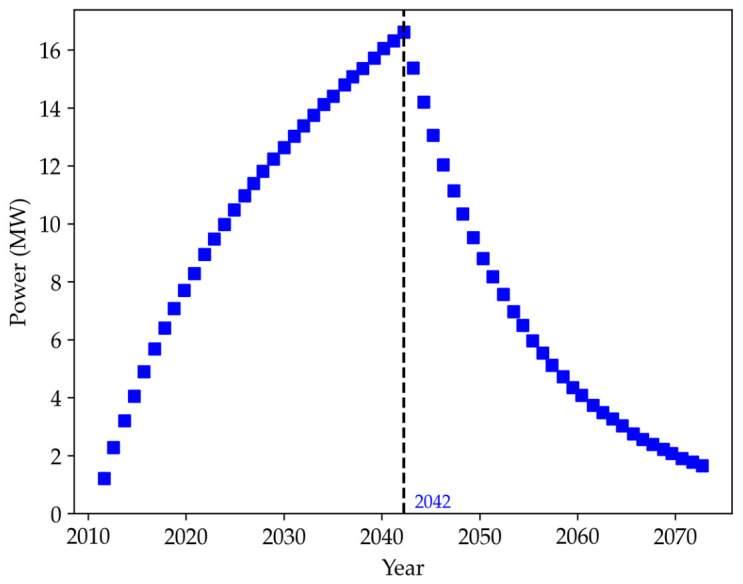
Estimated Electricity Generation Potential at the Maringá Sanitary Landfill.

For the generation of electricity in the landfill, the use of internal combustion engines was considered, with a utilization rate of 75% of the generated biogas, since part is oxidized and part is lost through the covering layer. Figure 5 shows the annual electricity generation profile for the landfill, producing a power of 16.517 MW, with maximum production in the year 2042.

As shown in Figure 5, the sanitary landfill in Maringá has a production capacity of approximately 1376 kW/month. Efficiency in electricity generation depends mainly on the biogas drainage system. For adequate generation, the Maringá landfill requires the installation of a structure for generating electricity on site, where it can take advantage of the biogas through a network of collector pipes that allow interconnection between the different areas of the landfill [38].

## 4. Discussion

### 4.1. Landfill Characteristics

This value is comparable to that reported for a state-of-the-art landfill located in Italy, which presents a value of 70 m^3^ per tonne of raw waste [39]. For mechanically–biologically treated waste, this value decreased to 20 m^3^/tonne, meaning a lower methane generation potential. Pheakdey et al. (2023) [40] found a value of 90 m^3^ CH_4_/tonne of MSW, which is slightly higher than the one considered in this study; however, this can be explained by the higher organic fraction of MSW considered by the authors. On the other hand, the L_0_ value obtained in this work was higher than those reported by Anh et al. (2021) [41] and Machado et al. (2009) [42], who conducted studies in landfills located in Nam Binh Duong (Vietnam) and Salvador (Brazil), respectively. Overall, the L_0_ value estimated in this study falls within the range reported by other authors. Therefore, monitoring the MSW composition throughout the landfill lifecycle is essential, as changes in waste treatment or in the habits of the population over time may alter the L_0_ parameter. For instance, a reduction in food waste (a major contributor to methane emissions) and an increase in plastic waste could ultimately reduce the L_0_ value [13].

From this perspective, variations in L_0_ among landfills may be associated with differences in the gravimetric composition of MSW, particularly the proportion of food waste and other easily degradable organic materials, as well as moisture availability and nutrient balance. Previous studies have shown that methane generation potential is directly related to the biodegradable organic fraction, whereas the decay rate constant is influenced by readily biodegradable content, moisture, temperature and carbon-to-nitrogen ratio (C/N) [43,44]. Hence, the L_0_ value adopted in this study suggests that the waste disposed of in the Maringá landfill retains a methane generation potential compatible with landfills where the organic fraction remains relevant.

The specific methane generation potential estimated for the landfill was approximately 0.06 tonne CH_4_ per tonne of waste, based on the L_0_ parameter derived from site-specific waste composition. This value is consistent with the range reported for municipal solid waste landfills in which, under conventional landfill conditions, methane yields typically range from approximately 0.04 to 0.07 tCH_4_/tonne MSW, depending on factors such as waste composition, moisture content, and operational practices, but higher ranges up to 0.14 tCH_4_/tonne MSW have also been reported when considering optimal biodegradation conditions [14,37,38].

The agreement between the present results and literature data suggests that the adopted modeling approach and input parameters provide a realistic representation of methane generation in the studied landfill. Moreover, the obtained values reflect typical conditions observed in developing countries, where waste heterogeneity and operational limitations influence biodegradation efficiency and gas recovery potential [4,13,14].

### 4.2. IPCC Model

Regarding the IPCC model results, the maximum annual methane production estimated for Scenario 2 (MCF 0.8) was 1.04 × 10^4^ tonnes CH_4_. The results obtained by Moghadam et al. (2021) [45] indicate that the maximum emissions in Iran were approximately 8 × 10^4^ tonnes CH_4_, a value similar to that found for C2.

Although Scenario 1 presents the highest annual methane generation, it is not consistent with the structure of the sanitary landfill in the municipality of Maringá—PR, as there is no adequate gas collection network, which could lead to biogas leakage and oxidation. According to the IPCC [46], sanitary landfills are classified as having appropriate conditions for anaerobic degradation, such as a depth of approximately 10 m, proper compaction, leachate collection systems, environmental monitoring of surface cover and rainwater, constant surface cover, and control of gas extraction and recovery of the generated gas.

Scenario 3 corresponds to an estimate for disposal sites that lack adequate operating conditions, such as dumps. Scenario 2 is the one that best suits the conditions of the city’s landfill, with an MCF of 0.8 used in the modeling, within the ranges suggested by the IPCC [46] of 0.3 to 0.8 for developing countries and tropical regions, such as Brazil. Furthermore, da Silva et al. (2020) [47] found a maximum methane generation of 1.61 × 10^4^ tonnes CH_4_, which is close to the one found in C2 of this study. One possible explanation for this similarity is that the authors conducted their study in Porto Alegre, Brazil, which presented similar climate and landfill operations to those considered in this case study. CH_4_ emission estimates vary depending on waste composition and operational practices [48]; however, these conditions can be realistically represented when using accurate data, as applied in this study. The model proves to be advantageous for designing gas collection systems in landfills across different regions [45].

The selection of Scenario 2 can also be interpreted from a process-based perspective. In landfills with intermediate management conditions, factors such as compaction, waste depth, cover practices, moisture retention, and drainage influence the formation of anaerobic microenvironments and, consequently, methane production [49]. In tropical landfills, these effects may be intensified by higher moisture and temperature, which can accelerate waste degradation when biodegradable organic matter is available [49,50]. Thus, the use of MCF = 0.8 appears more consistent with the local landfill conditions than either the assumption of a fully optimized sanitary landfill with complete gas recovery or the assumption of an unmanaged disposal site.

### 4.3. LandGEM Model

Regarding the LandGEM methane estimates, the total CH_4_ production ranged from 2.19 × 10^5^ to 2.28 × 10^5^ tonnes across the three scenarios. According to Moghadam et al. (2021) [45], the lowest production of CH_4_ was approximately 3.6 × 10^4^ tonnes, which is four times higher than the estimated production for C1, which is considered a well-managed landfill. This discrepancy may be attributed to differences in moisture content and the amount of organic compounds present. On the other hand, Bhar et al. (2025) [51] conducted a study on a landfill in Morocco and found a maximum methane production of 14.9 million m^3^, which is equivalent to 1.07 × 10^4^ tonnes CH_4_, much closer to the value found for C1 in this study. As a result, methane generation peaks vary due to the influence of MSW composition during the decomposition process. Organic materials are quickly degraded, accelerating gas production, whereas inert materials break down more slowly [47].

The CO_2_ emission value found for C2 in this study is very close to that reported by Nematollahi et al. (2024) [52] in their study describing gas emission in a landfill located in Iran. They found a maximum CO_2_ emission of 13.51 k tonnes/year. One factor that may explain this is the fact that the authors used the same value of k_2_ of 0.05, which corresponds to the default value recommended by the CAA. The slight difference in value may be due to the difference in L_0_, as the authors set a value of 170 m^3^/tonne, which is characteristic of arid and semi-arid regions.

After this time, production begins to decline due to the organic fraction that composes the city’s waste, which decomposes quickly, favoring the formation of biogas [47]. A study conducted in Southeast Brazil similarly found that, in the final year of landfill operations, the states of Minas Gerais, São Paulo, Rio de Janeiro, and Espírito Santo exhibited the highest methane generation rates, reaching 52.1%, 31.6%, 11.9%, and 4.4%, respectively [53]. This trend is linked to states with the highest MSW production, as well as a greater number of municipalities, further highlighting the potential of landfill methane as a renewable energy source. The LandGEM model presents higher production in Scenario 1, which considers landfills located in wetlands and bioreactors in which slurry, leachate, and other generated liquids are inserted in the process, to intensify the decomposition of waste [33]. For the analysis of this scenario, the k factor is not suitable for the analyzed landfill, because its structure does not fit into this context. Higher k values are generally associated with faster degradation of readily biodegradable organic matter, leading to earlier methane generation peaks, whereas lower k values indicate slower stabilization of the waste mass [43,44].

This parameter is, therefore, closely linked to waste composition, moisture, temperature and the availability of easily degradable organic substrates [44,54]. In tropical or subtropical regions, temperature and moisture conditions may accelerate biodegradation, but this effect depends strongly on local waste composition and landfill operation [49,50]. In this sense, the higher production estimated for Scenario 1 reflects the assumption of intensified waste decomposition under conditions that are not fully representative of the Maringá landfill, reinforcing the need to interpret LandGEM outputs according to site-specific operational conditions.

Scenarios 2 and 3 do not show significant differences in the production of biogas, methane and carbon dioxide. The distinction of the parameter k used is based on the criteria that each one was governed by, and k_2_ = 0.05 was based on the Clean Air Law (CAA), which regulates standards for sanitary landfills. On the other hand, the value of k_2_ = 0.04 was created based only on pollutant emission inventories. The similarity between these two scenarios suggests that, within the adopted parameter range, the model was less sensitive to small changes in k than to broader assumptions related to waste acceptance, L_0_, and operational conditions.

Considering the methane emission estimates obtained with both the IPCC and LandGEM models, the year 2042 showed two- to three-fold variations among the analyzed scenarios. The lower estimates obtained using the IPCC method may be attributed to the greater number of input parameters considered by this approach and to the classification of waste by category. In contrast, LandGEM, while practical, tends to overestimate gas generation because it may not adequately represent the climatic, operational, and waste-characteristic conditions of Brazilian landfills and does not explicitly consider gravimetric waste composition [47,55].

This difference can be further explained by the way each model represents landfill methane generation. While the IPCC method incorporates waste-specific parameters, LandGEM relies on simplified first-order decay parameters and does not explicitly account for gravimetric composition, moisture heterogeneity, methane oxidation in the cover layer, or spatial differences in landfill operation [39,56]. Previous studies have shown that default parameters may be inadequate for tropical landfills, where moisture, temperature, and organic waste content can differ substantially from the conditions under which many model defaults were developed. As a result, LandGEM may overestimate or misrepresent methane generation when default parameters are not calibrated for local conditions [56,57].

### 4.4. Measurement of Volume and Composition of Biogas

Regarding the biogas flow measurements, the higher production observed in drain 2 can be explained by the waste disposal time in the region where it is located. Similar results to drain 3 were obtained by Silva and Campos (2008) [58] when analyzing the biogas produced at the Bandeirantes—SP sanitary landfill, finding a maximum value of 3.67 × 10^2^ Nm^3^/h of biogas in one of the drains they evaluated.

The biogas flow values for drains 1 and 2 are lower compared to drain 3 (Figure 4c), since the cells (1 and 2) where they are located do not receive more waste and most of the values of the determined temperatures are below 30 °C, a condition that has a strong influence on the generation of landfill gas.

Among the various physical factors that influence the production of biogas, the temperature can be highlighted, which directly impacts microbial growth. Two optimal temperature ranges are considered for anaerobic digestion: 20 °C to 40 °C for mesophilic digestion and 40 °C to 60 °C for thermophilic digestion. However, for methanogenesis, mesophilic temperatures around 35–37 °C are frequently associated with higher methane productivity and greater process stability than thermophilic conditions [59,60]. Thus, the digestion and gasification process at temperatures close to or above 35 °C results in higher production, while at temperatures below 30 °C, there is a reduced level of gas production.

Other studies with different substrates also showed efficiency in the production of biogas under temperature conditions like those observed in this study. Souza et al. (2005) [61] analyzed residues from swine farming in different temperature ranges, noting that the best performance was at 35 °C. Silva and Abud (2016) [62] found that the biodigestion of sugarcane vinasse presented the best conditions under temperatures between 30 °C and 35 °C.

From a microbiological perspective, the lower biogas flow observed in some drains is consistent with the sensitivity of methanogenic activity to temperature. Temperatures below 30 °C tend to reduce microbial activity and slow the conversion of intermediate products into methane, whereas stable mesophilic conditions around 35–37 °C favor higher methane productivity and process stability [27,63]. Temperature may also affect the structure of methanogenic communities and the dominant metabolic pathways, helping to explain why gas production can vary within the same landfill when different cells present distinct thermal and degradation conditions [64,65]. Therefore, the lower temperatures observed in some landfill cells may have limited the activity of methanogenic consortia, contributing to the lower biogas flow measured in these drains.

The difference in the flow at the sampled points is due to the deposition time of each area, where points 1 and 2 had waste deposited longer and with activity already completed, while point 3 is located in an area where the disposal of waste is constant. According to Candiani and Torres (2015) [66], the generation of biogas results from several factors, including the waste degradation and engineering principles for structuring the landfill, such as the drainage system and operational procedures.

These differences among drains can therefore be interpreted as the combined result of waste age, degradation stage, temperature, moisture distribution, and gas collection efficiency. Older cells tend to contain a lower fraction of readily biodegradable organic matter, resulting in reduced methane generation rates, even when methane remains present in the gas phase [67,68]. In contrast, areas receiving more recent waste may still contain a higher amount of degradable substrate, favoring greater gas production. This interpretation is consistent with studies showing that fresh waste presents higher methane potential and faster decay rates, whereas aged waste shows depletion of degradable organic carbon [69].

Regarding the biogas composition, the average methane concentrations ranged from 51.10% (drain 1) to 57.06% (drain 3). These values are similar to those found in an experimental cell of a sanitary landfill, located in Recife—PE, in which average concentrations of 54.3 ± 2.7% of CH_4_, 40.7 ± 2.9% of CO_2_, and 1.2 ± 0.9% of O_2_ + N_2_ were obtained according to Maciel and Jucá (2011) [37], as well as the values determined by Penteado et al. (2012) [70] at the Paulínia—SP landfill, with methane concentrations between 51.9% and 59.3%.

Despite the three drains being at the landfill areas with very different disposal times, the biogas composition did not show a great difference for the analyzed compounds, including traces of H_2_S. Sulfur-containing compounds, even at low concentrations, can impair catalytic processes using biogas [71].

Despite the three drains being located in landfill areas with very different disposal times, the biogas composition did not show substantial differences for the analyzed compounds, including trace concentrations of H_2_S.

The detection of H_2_S has practical implications for landfill gas utilization and environmental monitoring [72]. From an energy perspective, sulfur-containing compounds can impair catalytic processes using biogas, cause corrosion, and reduce the performance of engines, catalysts, or upgrading systems, requiring gas cleaning before energetic use [71,73]. From a health and nuisance perspective, H_2_S and other odor-causing compounds have been associated with respiratory irritation, headaches, mucosal symptoms, and other complaints in populations living near landfills [74,75]. Therefore, this supports the need for integrated landfill gas management strategies that combine methane recovery and environmental monitoring.

### 4.5. Public Health and Sustainability Implications

Beyond its relevance for energy security and climate change mitigation, landfill gas management has important implications for public health. Methane emissions contribute indirectly to the formation of tropospheric ozone (O_3_), a pollutant associated with respiratory diseases and increased mortality. Additionally, trace gases such as H_2_S and NVMOCs may pose direct health risks, particularly for communities located near poorly managed landfills [5,6].

The methane concentrations observed in this study (up to 57%) indicate a high potential for controlled recovery. Without proper gas collection systems, these emissions would be released into the atmosphere, contributing not only to global warming but also to local air quality degradation. Therefore, implementing landfill biogas capture and utilization systems can significantly reduce population exposure to harmful compounds and improve environmental health and conditions.

From a broader public health perspective, the relevance of CH_4_ recovery extends beyond its role in ozone formation. Anthropogenic methane also contributes substantially to near-term warming, which is directly linked to increased heat-related mortality and greater cardiopulmonary risk. Staniaszek et al. (2022) [76] estimated that anthropogenic methane could account for approximately 690,000 premature deaths annually by 2050, both through ozone exposure and through about 1 °C of additional warming under a high-emissions scenario. Complementarily, Bell et al. (2024) [77] reviewed evidence indicating that extreme heat increases cardiovascular events, worsens respiratory conditions, and raises overall mortality risk. Taken together, these findings suggest that controlling methane emissions from landfill biogas may yield complementary health benefits by simultaneously reducing the ozone-related disease burden and limiting warming-driven health impacts.

This reasoning is linked to both CH_4_ and CO_2_ emissions. In the present study, simulations indicated that the landfill may emit up to 1.30 × 10^4^ tonnes of CH_4_ per year if the generated biogas is not recovered. Since methane is a potent greenhouse gas, its uncontrolled atmospheric release contributes significantly to long-term climate forcing and the associated public health burden. The energetic use of this biogas offers an important mitigation pathway, as it not only prevents direct CH_4_ emissions but also displaces fossil fuels otherwise required for electricity generation. Consequently, this substitution avoids additional CO_2_ emissions, which have been linked to global warming, environmental degradation, and increased risks of respiratory diseases and premature mortality. In line with the “mortality cost of carbon” proposed by Bressler (2021) [78], these findings reinforce that biogas recovery from landfills should be understood not only as an energy strategy but also as a climate and public health protection measure.

Considered together, these mechanisms strengthen the interpretation that landfill biogas capture can improve public health in two complementary ways: by limiting methane-driven ozone formation in the nearer term and by reducing cumulative CO_2_-related climate impacts over longer timescales.

Hence, the results discussed previously reinforce the importance of integrating landfill biogas recovery into public policies for solid waste management. Although Brazil has established regulatory instruments, such as the PNRS and economic incentives through the RenovaBio program, the practical implementation of energy recovery systems remains limited. In this context, initiatives such as the Methane Zero Program and international commitments under the Global Methane Pledge highlight the urgency of reducing methane emissions. The adoption of landfill biogas recovery technologies can contribute simultaneously to multiple Sustainable Development Goals (SDGs) from the United Nations (UN), particularly SDG 7 (Affordable and Clean Energy), SDG 13 (Climate Action), and SDG 3 (Good Health and Well-Being).

For developing countries where waste management infrastructure is often insufficient, landfill biogas recovery represents a cost-effective strategy to mitigate environmental pollution, reduce health risks, and promote sustainable urban development [79,80].

## 5. Conclusions

The results of this study demonstrated that the sanitary landfill in Maringá presents significant potential for biogas recovery and energy generation, supported by methane concentrations ranging from 51.10 ± 8.89% to 57.06 ± 1.19%. The application of different modeling approaches (IPCC, LandGEM, and CETESB) provided consistent estimates of methane generation, with projected production reaching up to 1.30 × 10^4^ tonnes of CH_4_ over a period of approximately 25–26 years. These findings confirm the technical feasibility of implementing gas capture and energy recovery systems in the studied site.

Beyond its energetic potential, landfill biogas recovery represents a strategic pathway for reducing environmental pollution and mitigating public health risks. The controlled capture of methane can significantly decrease greenhouse gas emissions and limit the formation of secondary pollutants such as tropospheric ozone, while also reducing the release of hazardous trace gases associated with adverse respiratory effects. These co-benefits are particularly relevant in developing countries, where inadequate waste management infrastructure often exacerbates environmental exposure and health vulnerabilities.

From a policy perspective, the results reinforce the importance of integrating biogas recovery into solid waste management frameworks and climate mitigation strategies. In the Brazilian context, such integration aligns with existing regulatory instruments and emerging initiatives aimed at methane reduction and renewable energy expansion. By contributing simultaneously to climate action, clean energy generation, and environmental health protection, landfill biogas recovery supports the advancement of Sustainable Development Goals, particularly SDG 3, SDG 7, and SDG 13.

Overall, this study highlighted landfill biogas recovery as a cost-effective and scalable solution capable of addressing interconnected challenges related to energy demand, environmental pollution and public health. Future efforts should focus on expanding the implementation of gas recovery systems in medium-sized municipalities and strengthening policy mechanisms to overcome current infrastructure and economic barriers. This study advances the current body of literature by integrating field-based measurements, scenario-driven modeling, and public health considerations into a unified assessment framework, offering a replicable approach for evaluating landfill biogas recovery in medium-sized municipalities of developing countries.

## Figures and Tables

**Table 1:** Fraction of degradable organic carbon by type of waste and methane correction factor.

Degradable Organic Carbon		Methane Correction Factor	
Components	DOC ^1^	Type of site	MCF ^2^
A—Paper and cardboard	40	Managed—anaerobic	1.0
B—Parks and gardens	17	Managed—semi-aerobic	0.5
C—Organic	15	Unmanaged—deep (>5 m waste)	0.8
D—Textile	40	Unmanaged—shallow (<5 m waste)	0.4
E—Wood	30	Uncategorized locations	0.6

^1^ DOC—Degradable Organic Carbon; ^2^ MCF—Methane Correction Factor. Source: IPCC, 2006 [31].

**Table 2:** Values of k_1_ for different residuals.

Paper	Organic	Textile	Wood	Gardens/Parks	Varied Domestics
0.07	0.4	0.07	0.035	0.17	0.05

Source: IPCC, 2006 [28].

**Table 3:** Values for methane generation potential (L_0_).

Landfill	Values of k_2_ (year^−1^)	Values of L_0_ (m^3^/tonne)
Conventional (CAA)	0.05 (default)	170 (default)
Arid Area	0.02	170
Conventional (Inventory)	0.04	100
Arid Area	0.02	100
Wet	0.7	96

Source: EPA, 2005 [33].

## Data Availability

Data will be made available upon request.
